# Production of norovirus-, rotavirus-, and enterovirus-like particles in insect cells is simplified by plasmid-based expression

**DOI:** 10.1038/s41598-024-65316-6

**Published:** 2024-06-27

**Authors:** Vili Lampinen, Stina Gröhn, Nina Lehmler, Minne Jartti, Vesa P. Hytönen, Maren Schubert, Minna M. Hankaniemi

**Affiliations:** 1 Virology and Vaccine Immunology, Faculty of Medicine and Health Technology, Tampere University, Tampere, Finland; 2 Department of Biotechnology, Institute for Biochemistry, Biotechnology and Bioinformatics, TU Braunschweig, Braunschweig, Germany; 3 Protein Dynamics, Faculty of Medicine and Health Technology, Tampere University, Tampere, Finland; 4 Fimlab Laboratories, Tampere, Finland

**Keywords:** Vaccine, Virus-like particle, VLP, Insect cells, Protein expression, Norovirus, Rotavirus, Enterovirus, Molecular biology, Expression systems, Nanobiotechnology, Recombinant vaccine, Proteins

## Abstract

Insect cells have long been the main expression host of many virus-like particles (VLP). VLPs resemble the respective viruses but are non-infectious. They are important in vaccine development and serve as safe model systems in virus research. Commonly, baculovirus expression vector system (BEVS) is used for VLP production. Here, we present an alternative, plasmid-based system for VLP expression, which offers distinct advantages: in contrast to BEVS, it avoids contamination by baculoviral particles and proteins, can maintain cell viability over the whole process, production of alphanodaviral particles will not be induced, and optimization of expression vectors and their ratios is simple. We compared the production of noro-, rota- and entero-VLP in the plasmid-based system to the standard process in BEVS. For noro- and entero-VLPs, similar yields could be achieved, whereas production of rota-VLP requires some further optimization. Nevertheless, in all cases, particles were formed, the expression process was simplified compared to BEVS and potential for the plasmid-based system was validated. This study demonstrates that plasmid-based transfection offers a viable option for production of noro-, rota- and entero-VLPs in insect cells.

## Introduction

VLPs are spontaneously assembled after expression of a single or several viral structural proteins. They resemble the complete three-dimensional shape of a virus without containing viral genetic information, which renders them immunogenic^1^, non-infectious and safe. Virus-like-particles (VLPs) are broadly utilized in vaccine development^2–4^, and in commercial vaccines, such as the human papillomavirus vaccines Gardasil^5^ and Cervarix^6^. Additionally, VLPs are a safe and practical model for studying the respective viruses. The baculovirus expression vector system (BEVS) in insect cells is one of the most common expression systems as it results in high protein yields^7,8^. Consequently, BEVS has been used to produce several recombinant-protein and VLP-based vaccine candidates, including candidates against influenza hemagglutinin (HA)^9^, Japanese encephalitis^10^ and Zika virus^11^. Up to date, more than 10 BEVS-derived vaccines are commercially available, including Cervarix™ (GSK, London, UK) against cervical cancer, Flublok® (Sanofi Pasteur, Paris, France) against influenza, and NVX-CoV2373 (Novavax, Malvern, PA, USA) against COVID-19^12^, highlighting the success of BEVS as a vaccine production platform.

Nevertheless, expression of VLPs by BEVS might be challenging: First, VLP production is always coupled to baculovirus production, resulting in baculoviral particles and proteins contaminating the produced VLP. Not only is separation of baculoviral particles during purification tedious or in some cases impossible due to their shared properties^13^ but the resulting VLP might also contain baculoviral proteins^14^ in its envelope. Second, BEVS is a lytic system, remodelling the host cell extensively during production, which might disrupt protein or VLP quality, especially in regard to secreted proteins^15–17^. Thirdly, High Five insect cells are known to be latently infected by alphanodavirus^18^. Baculovirus infection induces formation of alphanodaviral particles that contaminate the recombinantly produced VLP. VLPs that assemble from multiple different proteins require either co-infection or use of multicistronic or multipromoter baculoviruses. However, it has been demonstrated that co-infection is often limiting^19^ and the adaptation of protein ratios is mostly excluded^20^. To successfully produce several proteins at the same time, one baculovirus encoding multiple recombinant proteins can be employed^21^. This avoids the drawbacks of co-infecting with several baculoviruses. However, in general, generation, handling, titration and storing of baculovirus is laborious, entailing experience and time. Plasmid-based expression in insect cells presents an alternative for production of VLP^22–24^. It avoids the drawbacks described above for BEVS, but can still reach high yields of VLP, if lytic system is not required^22^.

Noro-, rota- and enteroviruses are enteric pathogens responsible for a variety of infections with substantial morbidity and mortality rates globally. In developing countries, noroviruses are the second leading cause of viral gastroenteritis in children under 5 years, and noroviruses are estimated to cause as many as 200,000 deaths annually among children in this age group^25^. After the introduction of widespread vaccination programs against rotavirus, norovirus has taken its place as the most common cause of viral gastroenteritis^26^. There are still no norovirus vaccines available in the market, but several vaccine candidates based on noro-VLPs have been developed by us^4,27,28^ and others^29^. Rotaviruses are among the most important agents of acute diarrhoea associated with mortality among children under five years old, as these viruses were estimated to cause approximately 453,000 deaths a year, mostly in countries of Africa and Asia^30^. Four oral live attenuated rotavirus vaccines are available internationally and they are considered effective in preventing severe gastrointestinal disease. However, in low-income countries, vaccine efficacy is lower than in industrialized settings, similar to other live oral vaccines^31^. Almost 300 human enterovirus (EV) serotypes cause a major impact on the public health. Out of all EVs, Coxsackie B enteroviruses (CVBs) are the most common cause of acute myocarditis, which is the major cause of sudden death in young adults^32^. 10–20% of the myocarditis cases progress to chronic dilated cardiomyopathy, which is a leading indication for cardiac transplantation^33^. Out of all human enteroviruses, only live weakened and inactivated forms of poliovirus vaccines are available globally^34,35^ and vaccines against EV71 are available in China and Thailand^36^. Therefore, new vaccines against enteroviruses as well as against noro- and rotaviruses would be urgently needed.

We have previously produced VLP-based vaccine candidates against norovirus^4,27,28^, rotavirus^2^ and coxsackie B3 (CVB3) enterovirus^37,38^ with BEVS. Here, we produced and analysed the noro-, rota- and CVB3 enterovirus VLPs (Noro-VLP, rota-VLP and CVB3-VLP) with plasmid-based insect cell expression and compared the production yields and the pure protein yields to those obtained with BEVS expression.

## Results

### Production and analysis of noro-VLP

**Figure 1:**
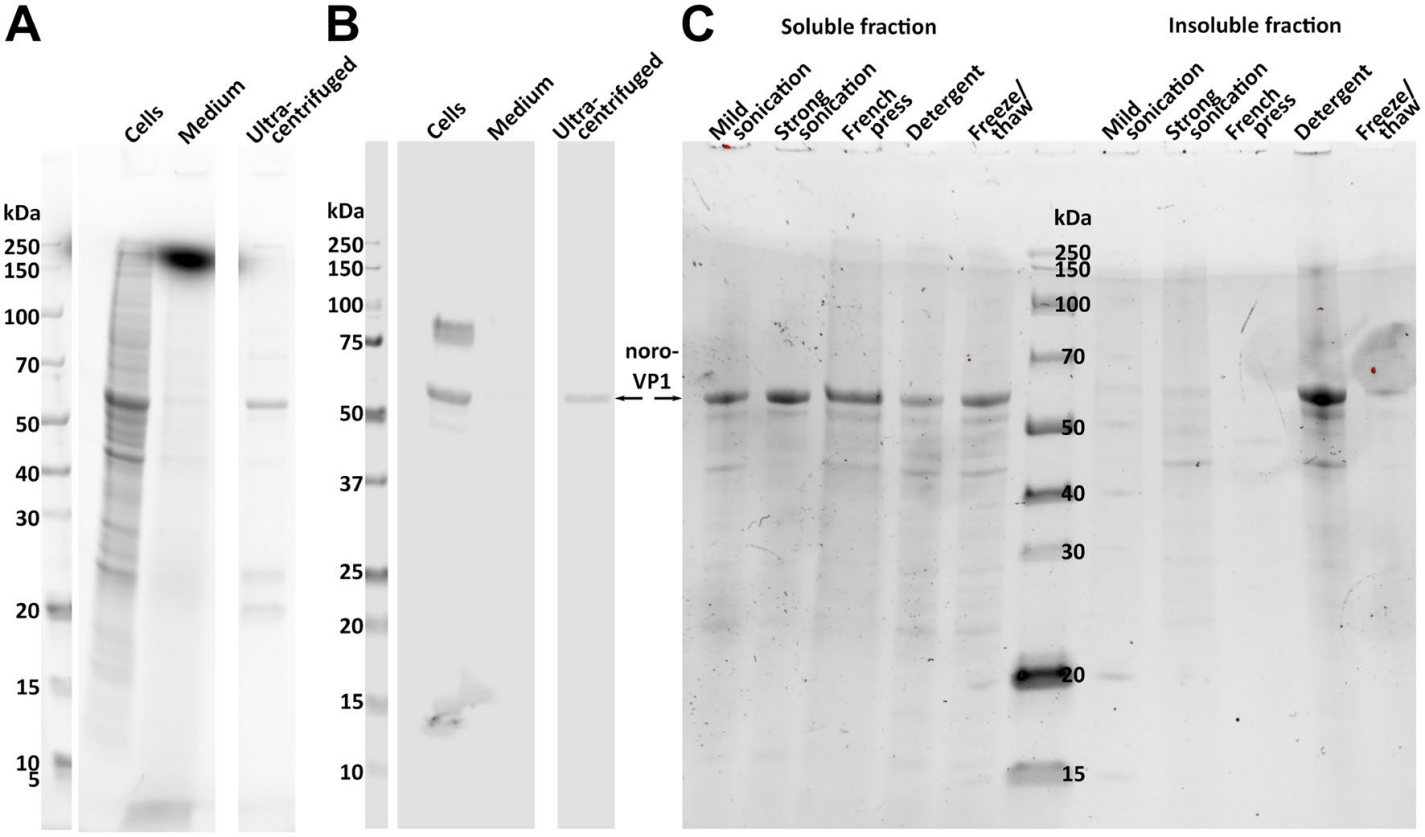
Optimization of plasmid-based noro-VLP expression. (**A**) Stain-free total protein staining of a test production of noro-VLP showing the cell fraction and a corresponding volume of medium after 5 days of expression. The third well has a sample of noro-VLP concentrated from the medium with ultracentrifugation. (**B**) A Western blot from the gel shown in A using an in-house polyclonal anti-noro-VLP antibody. (**C**) Stain-free total protein staining showing comparable samples of noro-VLP from a lysis method optimization experiment. The gels and blots were cropped for clarity. For full photos, see Supplementary Fig. 1.

We have previously used the BEVS insect cell system to produce modified and wild type noro-VLPs based on norovirus VP1 structural protein^39–41^. Here, we compared our BEVS insect cell expression method to plasmid-based expression in insect cells to see if similar yields and purities could be obtained without including baculovirus in wild type noro-VLP expression. When we have produced noro-VLP by using BEVS, noro-VLP can be observed almost exclusively in the extracellular fraction, so the most efficient way is to collect it from the production medium. However, five days after beginning plasmid-based expression, most of the norovirus VP1 was still found inside the cells (Fig. 1A,B), which were ~ 70% viable. We were able to extract and purify the intracellular noro-VLP at this time point after lysing the cells. Out of the methods tested, mild sonication resulted in most soluble noro-VLP released from the intracellular fraction (Fig. 1C). This resulted in a yield of 20 mg/L (assuming no changes after upscaling from 200 ml) of more than 95% pure noro-VLP determined by SDS-PAGE densitometry. By day 9, more than 95% of insect cells in the production were lysed and the vast majority of noro-VLP was released into the medium. We concentrated and purified noro-VLP from the medium using methods we have already established for BEVS insect cell expression^4^, and obtained a yield of 42 mg/L from a single comparable expression. We tried the same methods to purify extracellular noro-VLP after 5 days of plasmid-based expression, but only got 1.2 mg/L of noro-VLP at this point. At the same time, we produced batches of wild type noro-VLP with BEVS insect cell expression, collected them after 5 days from the production medium, and purified them using the same established methods. There, the yields were variable and between 1.9 and 35 mg/L with an average of 19 mg/L in these three batches. The yields are summarized in Table 1. It should be noted that with SpyTag-noro-VLP (differs from wild type only by a small C-terminal tag), which we have produced more frequently by BEVS, we have reached yields of up to 80 mg/L using the same methods, but also with variable results^4^.

**Table 1:** Yields of more than 95% pure noro-VLP by different expression methods. In BEVS, the amount of noro-VLP in the intracellular compartment is negligible, and we have established earlier that it is most efficient to collect noro-VLP from the production medium there.

Noro-VLP expression method	Culture length	Source	Yield (mg/l)
Transfected	5 days	Intracellular	20 ± 0.2
Transfected	5 days	Extracellular	1.2
Transfected	9 days	Extracellular	42
BEVS	5 days	Extracellular	19 ± 13

**Figure 2:**
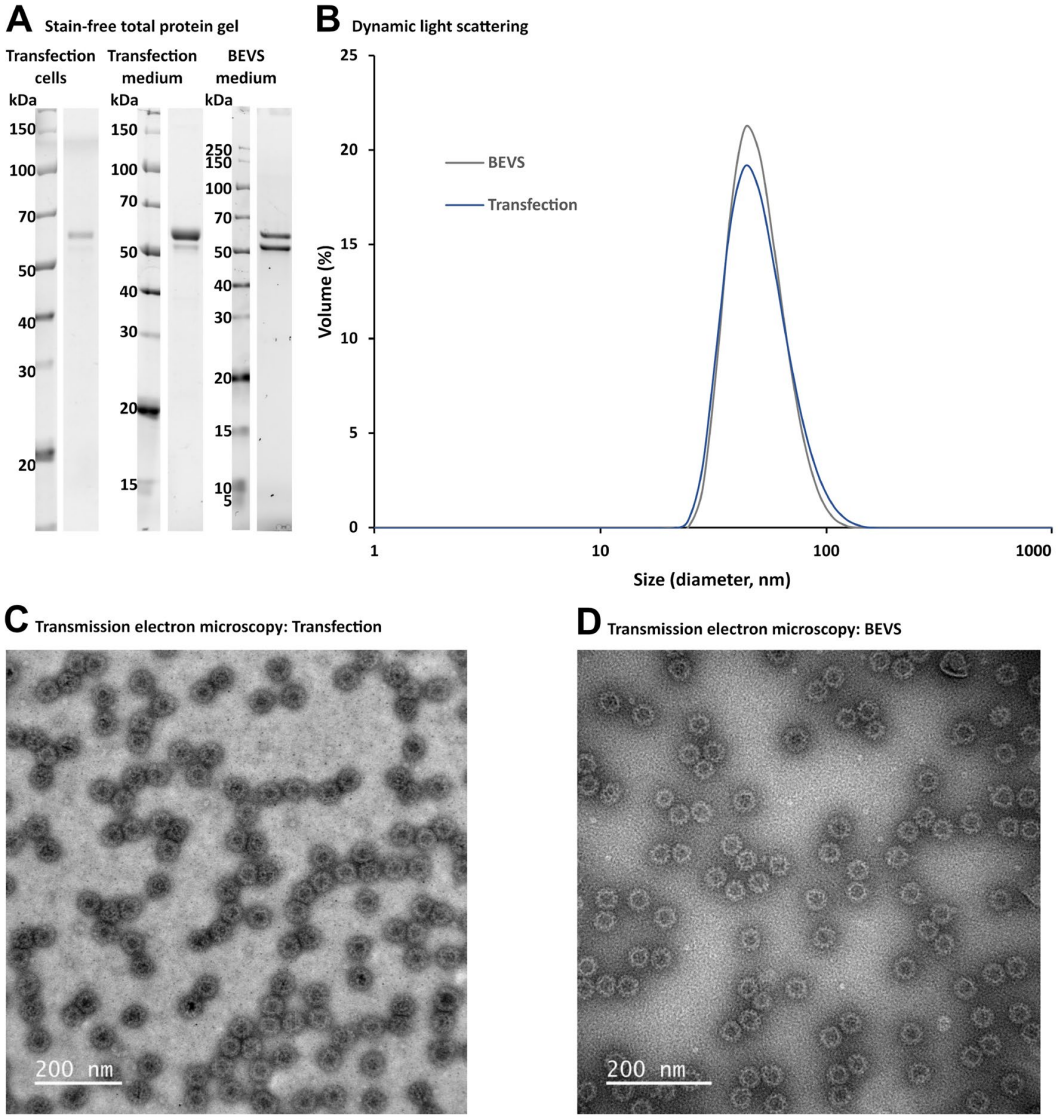
Characterisation of noro-VLP after purification. (**A**) Stain-free SDS-PAGE total protein analyses of noro-VLP purified from the cell (intracellular) or medium (extracellular) fraction of plasmid-based expression (transfection) or from BEVS expression medium. All three batches are from separate production batches prepared in the same way. The gels were cropped for clarity. For full photos, see Supplementary Fig. 2. In BEVS, noro-VP1 is known to appear as a double band due to an N-terminal proteolysis event^27^. (**B**) Dynamic light scattering analysis of the purified noro-VLP. (**C**) Transmission electron microscopy image of the purified noro-VLP from plasmid-based expression. (**D**) Transmission electron microscopy image of the purified noro-VLP from BEVS expression. Scale bars 200 nm, 60,000 × magnification.

We characterized the noro-VLPs obtained from BEVS and plasmid-based expression side by side. More than 95% of contaminating proteins were removed by ultracentrifugation and SEC, regardless of the expression method (Fig. 2A). Both methods produced particles with hydrodynamic diameters of approximately 50 nm based on dynamic light scattering (DLS) (Fig. 2B). In transmission electron microscopy (TEM), the particles seemed morphologically similar (Fig. 2C,D). When purifying noro-VLP from the intracellular fraction after 5 days of plasmid-based expression, noro-VLP was contaminated with intracellular DNA, which was not seen in other fractions. Removing the DNA was possible by including a DNase incubation step before the final purification step. In summary, plasmid-based expression resulted in identical noro-VLPs with yields in the same range as BEVS and with less batch-to-batch variation.

### Purification and analysis of rota-VLP

We have previously used BEVS insect cell system to produce rotavirus VP6 nanostructures as this production system is the traditional production system utilized for rota-VLPs^42^. Unfortunately, BEVS causes challenges in the purification of VP6 nanostructures. We have previously noticed that baculoviruses and VP6 proteins elute in the same fractions in anion exchange chromatography, and size exclusion chromatography is also challenging if the VP6 nanostructures are in their tubular form. Sucrose gradient ultracentrifugation has long been a gold standard in purifying VP6 nanostructures. This method, however, does not separate baculoviruses from VP6 nanostructures completely and a laborious pH-mediated process, which requires the dissociation and reassembly of VP6 nanostructures, is needed to disassemble and remove baculoviruses^43^. In this study, we produced VP6 nanostructures in High Five insect cells using traditional BEVS and a novel plasmid-based expression system to compare the yields and purities of the products and determine whether plasmid-based expression would be the solution to our purification challenges.

**Figure 3:**
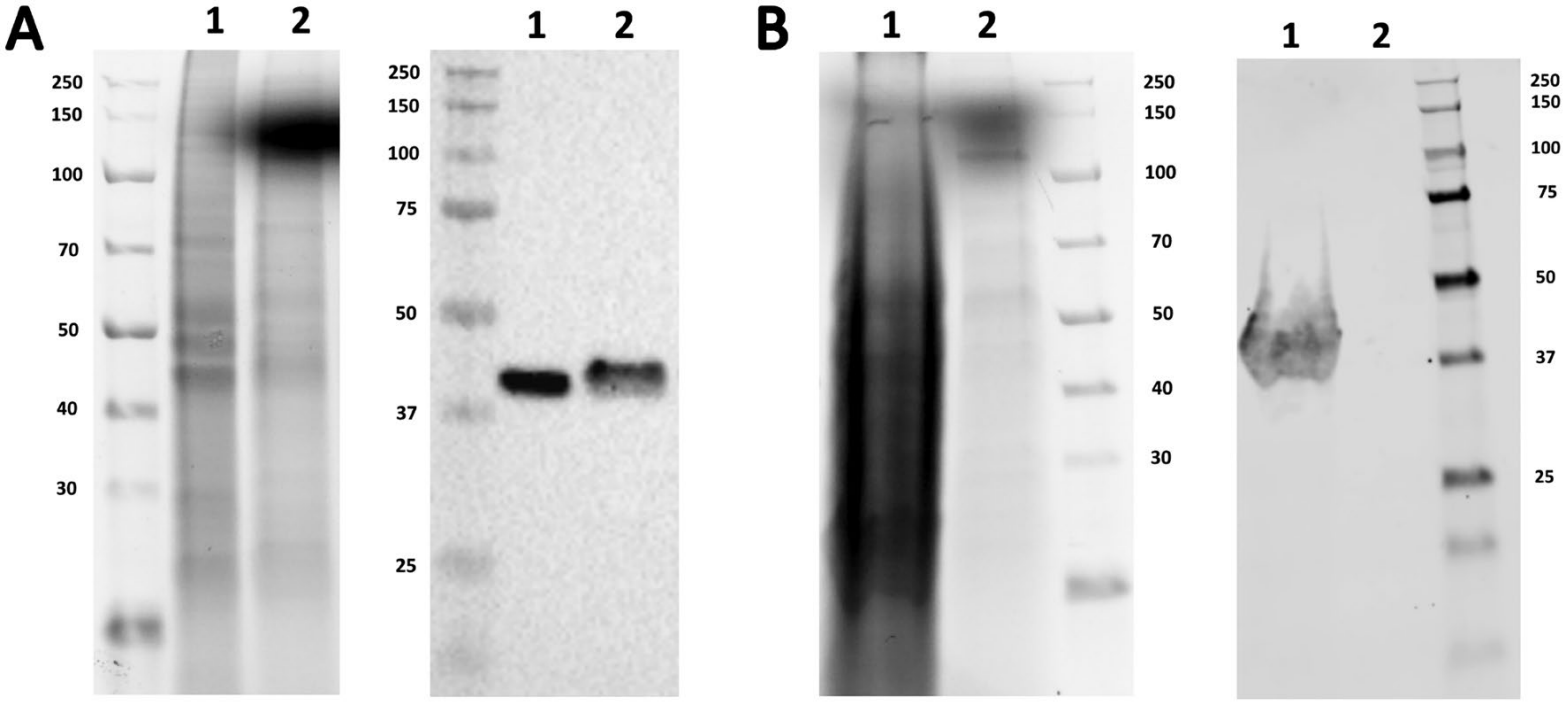
Accumulation compartments of VP6 produced in insect cells with BEVS or plasmid-base system. (**A**) SDS-PAGE and Western blot analyses of VP6 produced with BEVS. The left panel shows the stain-free total protein staining of the intracellular and extracellular samples from BEVS-based production. The right panel shows VP6 detection by Western blot using a commercial mouse VP6 IgG2a kappa monoclonal antibody. 15 µl of each sample was loaded per well as follows (1) intracellular BEVS-based sample and (2) extracellular BEVS-based sample. (**B**) SDS-PAGE and Western blot analyses of VP6 produced with plasmid-based system. The left panel shows the stain-free total protein staining of the intracellular and extracellular samples from plasmid-based production. The right panel shows VP6 detection by Western blot using a commercial mouse VP6 IgG2a kappa monoclonal antibody. 15 µl of each sample was loaded per well as follows: (1) intracellular plasmid-based sample and (2) extracellular plasmid-based sample. The gels and blots were cropped for clarity. For full photos, see Supplementary Fig. 3.

**Figure 4:**
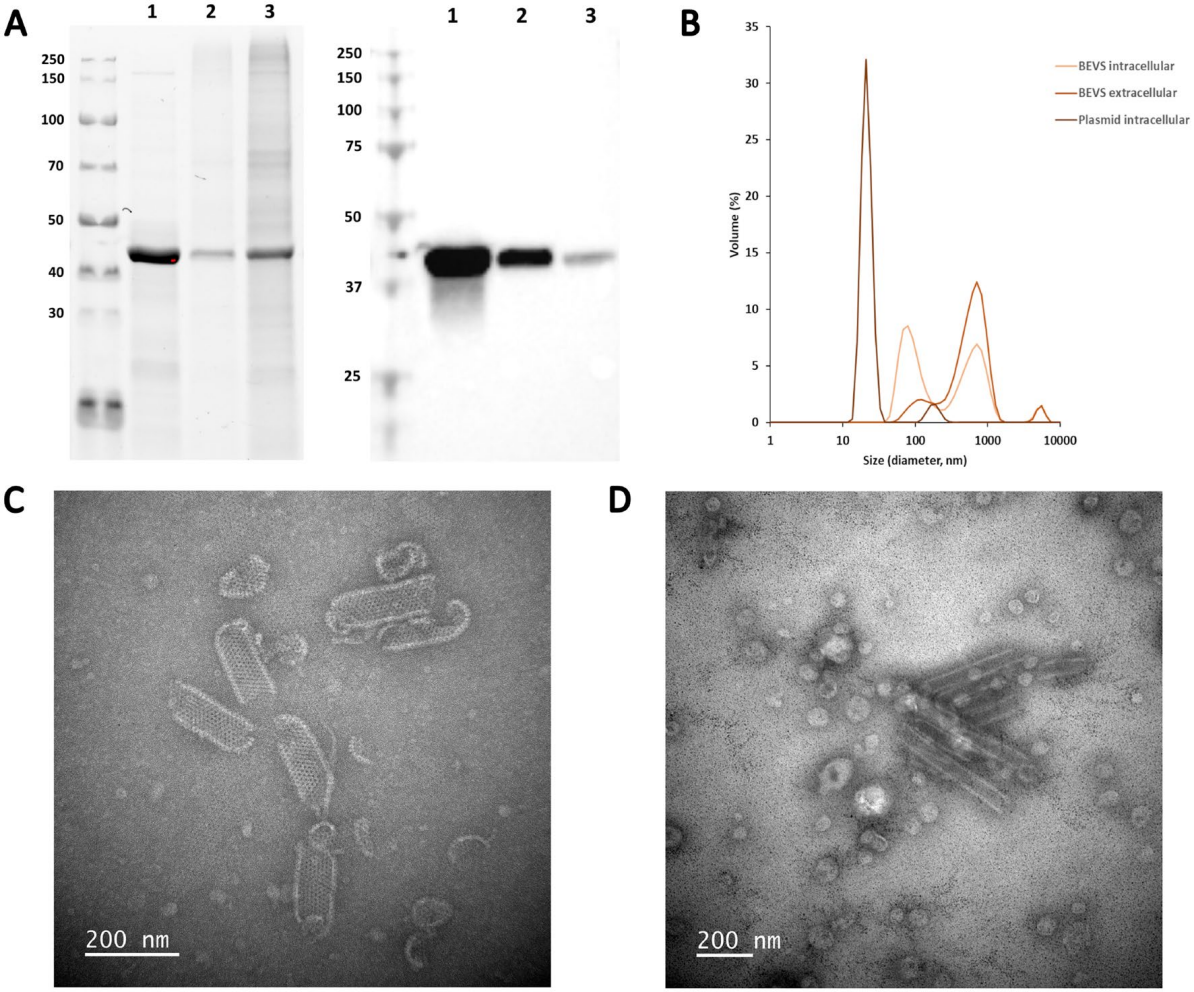
Characterisation of VP6 nanostructures after purification. A) SDS-PAGE and Western blot analyses of purified VP6s. The left panel shows the stain-free total protein staining of the purified VP6s. The right panel shows VP6 detection by Western blot using a commercial mouse VP6 IgG2a kappa monoclonal antibody. 15 µl of each purified VP6 was loaded per well as follows 1) Intracellular plasmid-based VP6 (c = 0.262 mg/ml, loaded 3.93µg) , 2) BEVS-based intracellular VP6 (c = 0.094 mg/ml, loaded 1.41µg) and 3) BEVS-based extracellular VP6 (c = 0.277 mg/ml, loaded 4.16µg). B) Dynamic light scattering analysis of the purified VP6s. C) Transmission electron microscopy (TEM) image of the sucrose gradient ultracentrifugation purified VP6 produced with plasmid-based system. Scale bar 200 nm, 50,000 × magnification. D) Representative TEM image of the sucrose gradient ultracentrifugation purified BEVS-based VP6. Scale bar 200 nm, 30,000 × magnification. The gels and blots were cropped for clarity. For full photos, see Supplementary Fig. 4.

First, we determined the accumulation locations of VP6 when the protein was produced with BEVS and plasmid-based systems. We collected both the extra- and intracellular protein fractions from 100 ml productions and analysed them with SDS-PAGE and VP6-specific Western blotting (Fig. 3). With BEVS, VP6 was found in both extra- and intracellular compartments (Fig. 3A). However, with plasmid-based expression system, VP6 was found only inside the cells, suggesting that VP6 needs the lytic effect of BEVS to be released into the production medium (Fig. 3B). Indeed, using BEVS approximately 90% of the cells were dead at the end of the cultivation whereas in the plasmid-based expression only less than 5% of the cells were observed to be dead. All the protein fractions were purified using ultracentrifugation and sucrose gradients. Sucrose gradient fractions were analysed by SDS-PAGE and VP6-specific Western blotting to identify the fractions containing VP6. Lastly, the fractions were combined, dialysed against PBS, and concentrated. SDS-PAGE analysis and the subsequent detection of the purified protein with a stain-free staining method (Fig. 4A) and Western blot analysis with anti-VP6 antibody (Fig. 4A) revealed a protein of approximately 45 kDa corresponding to VP6 in all purified products. According to densitometric analysis, the plasmid-based VP6 had a purity of 84%, the BEVS-based VP6 from the cell pellet 72% and the BEVS-based VP6 from production medium 40% (data not shown). Western blot analysis of the BEVS produced VP6 nanostructures with a commercial anti-gp64 antibody also revealed the presence of baculovirus still after purification (data not shown). The plasmid-based production yielded 170 µg pure VP6 from 100 ml culture (final concentration 0.262 mg/ml and volume 650 µl), which would mean approximately 1.7 mg of pure VP6 from one litre production. In turn, the BEVS production yielded roughly 15 mg/l (final concentration of VP6 purified from intracellular compartment 0.094 mg/ml and volume 1.4 ml, final concentration of VP6 purified from extracellular compartment 0.277 mg/ml and volume 5 ml). Although, when comparing the SDS-PAGE gel image and the blot of BEVS produced extracellular VP6 (Fig. 4A), it seems that the gel band also contains some other insect cell or baculovirus protein that is the same size as VP6, which distorts the densitometric analysis and extracellular yield. According to calculations, the second well in Fig. 4A contains 1.41 µg of VP6 and the third well 4.16 µg. Therefore, the VP6 band in the third well should be almost three times thicker than the VP6 band in the second well. However, the VP6 band is significantly thinner indicating that another protein of the same size is distorting the densitometric analysis and, thus, overestimating the extracellular yield.

VP6 forms the middle layer of the rotavirus capsid structure and, when expressed alone, VP6 is able to self-assemble into tubular or spherical nanostructures depending on the pH, ionic strength and divalent cation concentration^44^. In this study we analyzed the morphology of the VP6 nanostructures with DLS and TEM imaging. According to DLS analysis, 95% of the VP6 nanostructures from plasmid transfected cell pellet had an average hydrodynamic diameter of 22 nm and 5% had 181 nm (Fig. 4B). 47% of the VP6 purified from BEVS cell pellet had an average hydrodynamic diameter of 655 nm and 50% had 94 nm (Fig. 4B). Similarly, 84% of the VP6 purified from BEVS production medium had an average hydrodynamic diameter of 643 nm and 13% were 123 nm (Fig. 4B). The hydrodynamic diameter of approximately 650 nm obtained for BEVS produced VP6 nanostructures is in accordance with previously published results^2,45^. However, DLS is a challenging method for analysing tubular VP6 forms as it assumes the measured particles to be spherical. In the case of tubular VP6 nanostructures, the measured hydrodynamic diameter is only useful in comparing the batches of VP6 tubes to one another. In this study, the BEVS-based VP6 tubes purified from the cell pellet and production medium were similar in size, with a greater percentage of large tubes found in the VP6 structures purified from production medium. This is logical as the intracellular fraction might still contain VP6 tubes under construction. The VP6 tubes produced with plasmid-based system were smaller in size than the VP6 tubes produced with BEVS. The reason for this is currently unknown and pending further investigations, such as solving the VP6 structures with Cryo-EM.

Based on transmission electron microscopy (TEM) imaging, all the VP6 nanostructures had mostly tubular morphology (Fig. 4C,D), although, a few sphere-like structures were also seen. However, in BEVS produced batches these sphere-like structures are most likely some sort of vesicles or impurities, since they do not resemble VP6 spheres depicted in other studies ^45,46^. In the case of VP6 nanostructures produced with plasmid-based system, these sphere-like structures could be partially disbanded tubes or tubes still under construction, as the plasmid-based tubular structures seem shorter and less structured than BEVS-based VP6 tubes. Tubular observations are in accordance with literature, as according to Lepault et al. in chemical conditions like PBS VP6 nanostructures should have tubular form^44^.

### Production, purification, and characterization of CVB3-VLPs

We have previously produced CVB3 entero-VLPs using the Bac-to-Bac BEVS, where the P1 polyprotein was expressed under *polh* promoter and 3CD protease was expressed under *p10* promoter. Co-expression of CVB3 P1 and 3CD led to P1 polyprotein cleavage into VP0, VP1 and VP3 by 3CD and subsequent VLP assembly^37^. However, the production yields of the VLPs were moderate (~ 0.5 mg/l) and to enhance the VLP yield, we constructed a CVB3-VLP construct, in which the P1 polyprotein expression was under the strong *polh* promoter and 3CD protease under the weaker *CMV* promoter^38^. Additionally, we changed the baculoviral genome to flashBAC ULTRA, which contains deletions in the non-essential baculoviral protease genes^47^, thereby increasing the cell stability, and lowering the protease toxicity on the baculovirus-infected cells, allowing for a more efficient production of the target protein. In the new design, the 3CD protease was produced in low levels, which further decreases the toxic effects of 3CD on the host cells. In addition, we changed the production host cell from *Spodoptera frugiperda* Sf9 insect cells^37^ to *Trichoplusia Ni* High Five insect cells, which outperformed the Sf9 cells for VLP production yields^38^, as has been previously shown for the production of influenza-VLPs^48^. However, after all the improvements in the construct design and changing the production cell line, BEVS system causes complexity that might be prevented with plasmid-based insect cell expression. Therefore, here, we tested the feasibility of co-transfecting two monocistronic plasmids (plasmids encoding 3CD protease or P1 capsid polyprotein) in insect cells and compared the pure VLP yields on those obtained after expressing the CVB3-VLP utilizing single polycistronic baculovirus (flashBAC ULTRA baculoviral genome)^38^.

First, we determined the effect of 3CD/P1 co-transfection ratio on the CVB3-VLP production level and particle morphology. High Five cells were transfected with 2%, 5% or 10% 3CD protease plasmid ratio in proportion to P1 polyprotein plasmid and 5- and 8-day productions were collected. We collected extra- and intracellular VLP-protein fractions from 40 ml productions, clarified the production medium by sterile filtration and lysed the cells by sonication (and clarified the released intracellular VLPs with sterile filtration). The intra- and extracellular fractions were concentrated with 30% sucrose cushion ultracentrifugation as we have done previously^49^.The yields from the intracellular VLP fraction were negligible (data not shown). This is consistent with our previous findings (unpublished). The yields from the extracellular VLP fractions were between 1.3 and 2.1 mg pure VLP from 40 ml culture (Table 2.), which would correspond to roughly 30–50 mg from one litre production.

**Table 2:** Pure CVB3-VLP yields after 30% sucrose cushion ultracentrifugation of the extracellular protein fraction (from cleared supernatant).

CVB3-VLP production details	Culture length	Yield from 40 ml (mg)
2% 3CD/98% P1	5 days	1.6
	8 days	1.9
5% 3CD/95% P1	5 days	1.8
	8 days	2.1
10% 3CD/90% P1	5 days	1.3
	8 days	1.3

**Figure 5:**
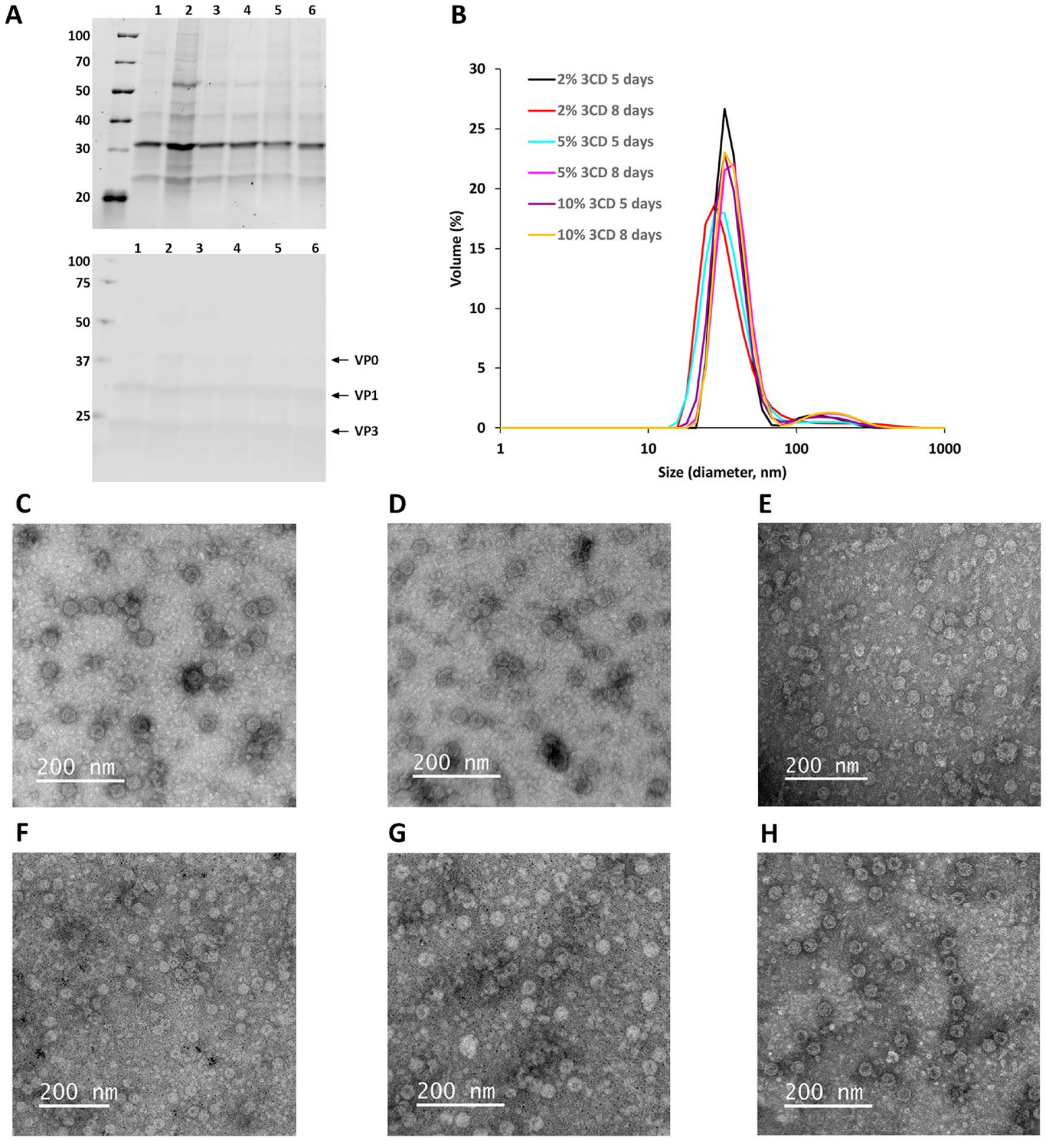
Characterisation of CVB3-VLPs produced by co-transfection of 3CD and P1. (**A**) SDS-PAGE and Western blot analyses of purified VLPs. The top panel shows the stain-free total protein staining of the purified CVB3-VLPs. The bottom panel shows VP0, VP1 and VP3 capsid protein detection by Western blot using an in-house produced rabbit anti-CVB1–6 polyclonal antibody. 2 µg of each purified CVB3-VLP was loaded per well recovered from cultures produced as follows (1) 2% 3CD/98% P1, 5 days production, (2) 2% 3CD/98% P1, 8 days production (3) 5% 3CD/95% P1, 5 days production, (4) 5% 3CD/95% P1, 8 days production (5) 10% 3CD/90% P1, 5 days production, 6) 10% 3CD/90% P1, 8 days production. The gel and blot were cropped for clarity. For full photos, see Supplementary Fig. 5. (**B**) Dynamic light scattering analysis of the purified CVB3-VLPs. Transmission electron microscopy images of the sucrose cushion ultracentrifugation purified CVB3-VLPs: (**C**) 2% 3CD/98% P1, 5 days production, (**D**) 2% 3CD/98% P1, 8 days production (**E**) 5% 3CD/95% P1, 5 days production, (**F**) 5% 3CD/95% P1, 8 days production (**G**) 10% 3CD/90% P1, 5 days production, (**H**) 10% 3CD/90% P1, 8 days production Scale bar 200 nm, 50,000 × magnification.

SDS-PAGE analysis and the subsequent detection of the purified proteins with a stain-free staining method (Fig. 5A, top panel) and Western blot analysis (Fig. 5A, bottom panel) revealed proteins of approximately 38 kDa, 31 kDa, and 26 kDa corresponding to the CVB3 capsid proteins VP0, VP1 and VP3. Based on DLS, 92–95% of the CVB3-VLP particles had an average hydrodynamic diameter of ~ 35 nm (Fig. 5B) and according to TEM imaging, the particles had the expected spherical morphology (Fig. 5C–H).

To be able to compare the pure protein yields from VLPs produced with BEVS versus plasmid-based transfection system, we made a 200 ml CVB3-VLP production by co-transfecting 5% 3CD and 95% P1 plasmids and harvesting the culture supernatant after 8 days in culture. We did not do thorough comparison of the repeatability of the expression levels between different CVB3-VLP expression conditions as the pure protein yields did not vary significantly between the conditions used. We concluded that any of the chosen conditions can be used in plasmid-based CVB3-VLP expression and we chose these plasmid ratios and production length as the pure protein yield was the highest (2.1 mg) in this condition in the 40 ml culture.

**Figure 6:**
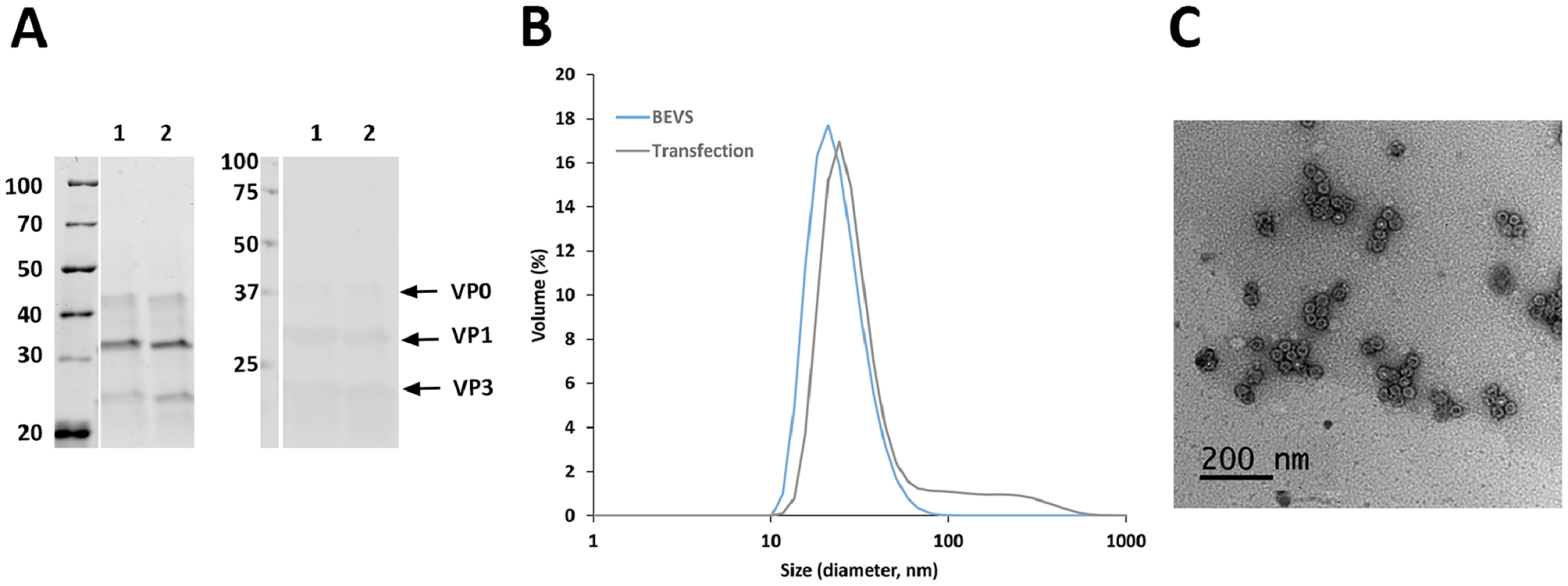
Characterisation of CVB3-VLP after pilot-scale production and ion-exchange purification. (**A**) SDS-PAGE and Western blot analyses of the purified VLPs. The left panel shows the total protein staining of the purified CVB3-VLPs with stain-free method. The right panel shows VP0, VP1 and VP3 capsid protein detection by Western blot using an in-house produced rabbit anti-CVB1-6 polyclonal antibody. 2 µg purified CVB3-VLP was loaded per well produced as follows (1) 10% 3CD/90% P1, 8 days production, (2) BEVS, 5 days production. The gel and blot were cropped for clarity. For full photos, see Supplementary Fig. 6. (**B**) Dynamic light scattering analysis of the purified CVB3-VLPs from BEVS or plasmid-based production followed by tangential flow filtration and ion exchange chromatography purification (**C**) representative transmission electron microscopy image of purified CVB3-VLP from plasmid-based expression system. 50,000 × magnification, scale bar 200 nm.

For comparison, the 200 ml culture supernatant was purified with the same BEVS-based protocol we have previously developed for CVB3-VLP^38^. Briefly, the VLP was concentrated by tangential flow filtration (TFF), utilizing 750 molecular weight cut-off (MWCO) hollow fibre with an ÄKTA Flux system. The buffer was exchanged to 40 mM Tris pH 7.3, 10 mM MgCl_2_, 40 mM NaCl, and 0.1% Tween80 with the same system, and the impurities were removed with anion exchange chromatography and finally the VLPs were captured into SO_3_ cation exchange chromatography column and were eluted using NaCl gradient. The final yield of the pure CVB3-VLP was 0.3 mg from 200 ml production, which would correspond to 1.5 mg from 1 L production. This is exactly the same pure protein yield as we have previously reported for CVB3-VLP after BEVS production in the same cell line and purified with the same purification protocol^38^. SDS-PAGE analysis and the subsequent detection of the proteins with a stain-free staining method (Fig. 6A, left panel) and Western blot analysis (Fig. 6A, right panel) revealed proteins of approximately 38 kDa, 31 kDa, and 26 kDa corresponding to the CVB3 capsid proteins VP0, VP1 and VP3. According to DLS analysis, 94% of the CVB3-VLP particles produced with plasmid-based system had an average hydrodynamic diameter of 32 nm, whereas 100% of the CVB3-VLP particles we have produced previously with BEVS^38^ had an average hydrodynamic diameter of 30 nm (Fig. 6B). According to TEM imaging, particles produced with both systems had spherical morphology and representative CVB3-VLP TEM image from large scale production and purification is shown in Fig. 6C.

## Discussion

In our earlier work, we have used the different methods of BEVS for VLP expression in insect cells^3,4,28,38,50^. Consequently, we have also developed several purification methods for being able to separate the different VLPs from the baculovirus and host cell contaminants effectively. Although size-exclusion chromatography (SEC) -based separation provides excellent yields of noro-VLP with good separation from residual baculovirus^4^, the method is difficult to scale up. When we used a more scalable anion exchange chromatography (AEX) method, the yield was lowered by partial overlapping of baculovirus and noro-VLP elution conditions^28^. In our experience, the same purification methods and parameters are usually not applicable between different VLPs, so each VLP modification requires optimization of the purification pipeline. The optimization is more difficult when you have to maintain conditions that also separate baculovirus efficiently from your target VLP. Therefore, VLP expression without baculoviruses would be highly desirable. In this study, we experimented plasmid-based insect cell expression in production of noro-, rota-, and CVB3-VLPs.

We prepared the noro-VLP plasmid for plasmid-based expression and made test productions according to earlier publications with the method^24,51^. However, we noticed that after 5 days of expression, most noro-VLP resides inside the insect cells. However, with optimal lysis conditions, we obtained pure noro-VLP with similar yields as compared to BEVS expression. Then again, collecting VLP from cells was more complicated and time-consuming than collection from production medium. In BEVS, the noro-VLP is presumably released from the cells only after cell lysis by baculovirus 3–6 days after infection. In plasmid-based expression, the cells seem viable for a longer time^24^ and lyse completely only after 8 or 9 days. By increasing expression time to 9 days, we obtained a similar/higher yield of noro-VLP from the supernatant (42 mg/l) with the same methods that we utilize for BEVS expression of noro-VLP (up to 35 mg/l). It should be noted that yields are extrapolations from smaller production volumes and higher culture volumes may need more scalable and complex purification methods in practice, which may affect VLP yields. With the methods used, the final product was undistinguishable from noro-VLP produced with baculoviruses.

Rotavirus VP6 structures were also successfully produced with plasmid-based expression system. Nevertheless, the production process is still in need of optimization. The yield with plasmid-based system was much lower than with the traditional BEVS, although the purity was higher. Cultivation time for plasmid-based expression was 5 days, when with BEVS it was 7 days. The plasmid-based expression could potentially benefit from increasing the number of cultivation days. According to TEM imaging, all the VP6 structures had mostly tubular morphology, though a few sphere-like structures were also seen among the VP6 nanostructures. Rotavirus VP6 has the ability to self-assemble into tubular or spherical nanostructures depending on the pH, ionic strength and divalent cation concentration, when expressed without other rotavirus capsid proteins^44^. The plasmid-based sphere-like structures observed in this study could be partially disbanded tubes or tubes still under construction, as the plasmid-based tubular structures seemed shorter and less structured than BEVS-based VP6 tubes. In the case of BEVS produced batches the observed sphere-like structures are mostly likely some sort of vesicles or impurities, since they do not resemble VP6 spheres depicted in other studies^45,46^. Previously, Lepault et al. observed tubular VP6 nanostructures in chemical conditions like PBS^44^. According to DLS analysis, the plasmid-based VP6 structures were smaller in size than the BEVS-based VP6 structures and even smaller than those observed in the TEM image. DLS data analysis used here assumes the measured particles to be spherical, so the measured hydrodynamic diameter is only useful in comparing the batches of VP6 tubes to one another in the case of tubular rota-VLP. The BEVS-based VP6 tubes purified from the cell pellet and production medium were similar in size, with a greater percentage of large tubes found in the VP6 structures purified from production medium. This is logical as the intracellular fraction might still contain VP6 tubes under construction. The observed hydrodynamic diameters of approximately 650 nm are, also, in accordance with previous literature^2,45^. Further studies are needed to determine the reasons on why and how the plasmid-produced and BEVS produced VP6 nanostructures are different. For example, Cryo-EM could be used to solve the atomic structures of both of these nanostructures.

Finally, also CVB3-VLP was successfully produced with plasmid-based system and the ratio of 3CD protease/P1 polyprotein did not cause significant effect on the production yield of the VLP. This demonstrates that co-transfection of the plasmid encoding the P1 polyprotein and 3CD polyprotein worked very well. When CVB3-VLP was produced in 200 ml volume and it was purified with the same protocol we have previously developed for CVB3-VLP^38^, the obtained yield after purification was approximately 1.5 mg/l, which is the same yield as that obtained for CVB3-VLP produced with BEVS in our previous study^38^.

In general, we could show that plasmid-based expression of VLPs is a valid alternative to BEVS with at least similar production yields for two out of our three tested VLP species. Co-transfection of more than one plasmid to e.g. produce CVB3-VLPs, which require procession of 3CD protease, worked and ratios can be adjusted to the most optimal outcome. For mammalian cells, it is described that more than 100,000 plasmids can enter one cell^22^, which can be assumed to be applicable to other eukaryotic cells as well. We could show that 5% 3CD protease plasmid was enough to process the polyprotein efficiently (Fig. 4A), indicating that statistically all cells had been transfected with at least one 3CD protease plasmid at this ratio. In addition, it only took 5–9 days from the transfection to harvest the VLPs, compared to approximately one month needed to generate recombinant baculovirus with the flashBAC method, amplify the virus stocks, titrate the virus, and finally produce VLP by BEVS. Amplification of baculovirus in insect cells is not only significantly slower than plasmid amplification in *E. coli* for plasmid-based expression, but LB broth is also inexpensive compared to insect cell medium. Material cost differences may not be decisive in the experimental setting but gain significance when moving to the industrial scale. In all cases the VLP purification was simpler and no contamination by baculoviral or alphanodaviral particles is expected^18^ nor was observed in TEM, DLS or PAGE. Even contamination by extracellular vesicles is reported to be lower in transfected *Trichoplusia ni* insect cells, as compared to baculovirus-infected cells^52^. In this work, we did not compare production of enveloped VLPs, whose expression with the plasmid-based insect cell expression system has been validated^22^. In enveloped VLPs, we expect the difference of the two expression systems to be even more pronounced, as enveloped recombinant VLP produced by BEVS will always integrate overexpressed baculoviral proteins like gp64 in the VLP^53^. In conclusion, the plasmid-based expression led to a faster production and simpler purification of VLPs while still enabling high yields.

## Material and methods

### Expression vector construction

For baculovirus-based expression, the genes of norovirus capsid protein VP1 from strain Hu/GII.4/Sydney/NSW0514/2012/AU (GenBank ID: AFV08795), rotavirus inner capsid protein VP6 (GenBank ID: ACL93331.1), the enteroviral polyprotein VP1–VP4 (GeneID M33854.1), and the 3CD protease (GeneID M33854.1) were synthesized (Genscript, USA) and cloned into pOET5 baculovirus transfer vectors under the polyhedrin promoter with the exception that 3CD protease was cloned under *CMV* promoter control, creating a dual promoter construct. We used these pOET5 plasmids as templates and subcloned the genes into the OpiE2 expression vector^54^ for plasmid-based expression. There, we inserted them after a 5′UTR Kozak sequence validated before^24^. For plasmid-based expression, the enteroviral VP1–VP4 polyprotein and the 3CD protease were subcloned into separate plasmids. Sequences were confirmed by sequencing and plasmid DNA prepared using NucleoBound Xtra Midi-Kit (Macherey-Nagel, Germany) according to the manufacturer’s protocol.

### Plasmid-based expression in insect cells

Expression of the VLP was performed in High Five cells (Thermo Fisher Scientific, USA), as described before^51^. Notably, High Five cells had been passaged over 100 times, linear 40 kDa Polyethylenimin (PEI) (PolyScience, USA) and EX-CELL 405 serum-free medium (Sigma-Aldrich, USA) was used for production, all factors important for optimal production. Cells were transfected with 1 µg DNA/million cells using plasmid purified by a standard purification kit (NucleoSpin Plasmid EasyPure Kit NucleoBond Xtra Midi Kit, A260/A280 ratio between 1.8–2.0, concentration between ~ 300–1000 ng/µL). Starting cell density was 4 million cells/ml, multiplied eightfold in volume during cultivation, and harvested after 96–192 h (4–8 days) of expression by centrifugation for 4 min at 180 g. Supernatant was harvested 96–192 h after transfection by a two-step centrifugation: First, 4 min at 180 g to remove cells while not risking cell disruption, followed by 10 min at 1000 g to remove cell debris.

### Baculovirus-based expression in insect cells

Recombinant baculovirus stock was generated using flashBAC ULTRA baculovirus kit (Oxford Expression Technologies, UK) according to manufacturer’s instructions. Briefly, recombinant baculovirus was generated by mixing the baculovirus DNA from the kit with the pOET5 vector containing the genes that encode noro-, rota-, or enteroviral proteins and baculoFECTIN II transfection reagent and transfecting *Spodoptera frugiperda* cells (Sf9, Thermo Fisher Scientific) with the mixture. After five days, the P0 baculovirus stock was collected. The P0 stock was used to infect Sf9 cells (2 × 10^6^ cells/ml) to generate P1 stock and the P1 stock to generate P2 stock. The titres of the baculovirus stocks were determined using a BacPAK™ Baculovirus Rapid Titer Kit (TaKaRa, USA). The VLPs were produced in High Five cells in Insect-XPRESS™ Protein-free Insect Cell Medium (Lonza, USA) using the P2 stock, multiplicity of infection (MOI) value of 1 pfu/cell for rota- and CVB3-VLPs and 10 for noro-VLPs, cell density of 2 × 10^6^ cells/ml and cultivation time of 4–7 days. The cultured cells and supernatant were harvested by centrifugation (1000 g at + 4 °C for 30 min).

### Purification and characterization of noro-VLPs

After plasmid-based expression in 50 or 200 ml of High Five cell suspension, the produced noro-VLP was harvested either from the cell pellet or in released form from the supernatant. When harvesting noro-VLP from the supernatant in plasmid-based or BEVS expression, we purified it as described earlier for baculovirus-derived VLPs^4^. It was first concentrated into a pellet by ultracentrifugation (175,000 g, 6–16 h). The noro-VLP pellet was then dissolved in PBS and purified in size-exclusion chromatography using ÄKTA Purifier instrument and HiPrep 16/60 Sephacryl S-500 HR column (Cytiva, USA). For storage, noro-VLP was concentrated to more than 1 mg/ml with VivaSpin Turbo spin tubes (10,000 MWCO, Sartorius, Germany) and sterile-filtered.

When harvesting from inside the insect cells, the cells first needed to be lysed gently to release noro-VLP in soluble form. After resuspending the cell pellets from parallel 50 mL production runs into 5 ml PBS or lysis detergent (40 ml PBS to do French press), we tried repeated freeze/thaw cycles, I-PER insect cell lysis detergent (Thermo Fisher Scientific), French press and sonication. A 2-min sonication with cycles of 2 s pulses followed by 5 s off time at an amplitude of 40% worked best in releasing noro-VLP in soluble form. The lysate was centrifuged (47 000 g, 15 min) and filtered through 0.2 µm syringe filter to clarify and to separate soluble proteins from insoluble proteins and cell fragments. After this, noro-VLP was purified from the soluble fraction by size-exclusion chromatography, as explained above. Densitometric analysis of all purified VLP products was performed with ImageLab™ software (version 6.0.1. build 34 Standard Edition, Bio-Rad) or ImageJ^55^ after separation of proteins by SDS-PAGE using Mini Protean TGX Precast gels (Bio-Rad Laboratories, USA). To verify the presence of noro-VP1, the proteins separated by SDS-PAGE were transferred onto a nitrocellulose membrane (Bio-Rad) using the Trans-Blot® Turbo™ Transfer System for western blotting. The membranes were immunoblotted using 1:2000-diluted in-house polyclonal anti-noro-VLP antibody and 1:20,000-diluted IRDye 800CW goat anti-mouse IgG (LI-COR Biosciences, USA). The fluorescent bands were visualized with the Odyssey instrument (LI-COR Biosciences). Determination of VLP total protein concentration by BCA, dynamic light scattering (DLS) analysis, and TEM imaging were performed as previously^38^.

### Purification and characterization of rota-VLPs

Rotavirus inner capsid protein VP6 was produced in High Five insect cells utilizing both the plasmid-based expression system and BEVS. The purification and characterization were performed similarly for both expression methods. Extra- and intracellular VP6 protein fractions were collected from 100 ml productions by pelleting the cells and clarifying the production medium. At first, the pellets were dissolved in 2 ml of 20 mM Tris buffer pH 7.5 and lysed by freeze/thawing. The freeze/thaw cycle was performed three times and during each cycle 2 ml of the previously mentioned Tris buffer was added to the suspension. After the cell lysis, the cell debris was removed by centrifugation at 10,000 g for 15 min at 4 °C. The clarified cell lysates were then ultracentrifuged at 100,000 × g for 1.5 h at 4 °C and the resulting protein pellets were resuspended in 0.2 M Tris buffer pH 7.5 and incubated overnight at 4 °C. The resuspended pellets were laid on top of “continuous” sucrose gradients consisting of seven layers (10–70%) and centrifuged at 100,000 g for three hours at 4 °C. After centrifugation, fractions were collected through bottom puncture. The fractions were analysed by SDS-PAGE and by VP6-specific Western blotting to verify the presence of VP6, as described above. . The western blot membranes were immunoblotted using 1:3000-diluted monoclonal mouse rotavirus VP6 antibody IgG2a kappa (Novus Biologicals, USA) and 1:10,000-diluted horse anti-mouse IgG-HRP (Vector Laboratories, USA). The immunoblots were developed using WesternBright ECL kit (Advansta, USA). Fractions containing the desired VP6 protein were combined and dialyzed against sterile PBS overnight using Slide-A-Lyzer Dialysis Cassette 10,000 MWCO (Thermo Fisher Scientific). Finally, the protein products were concentrated using Amicon® Ultra-15 Centrifugal Filter Units (Ultracel-30 kDa cutoff) (Merck Millipore, Ireland). After purification, the presence of baculovirus was analysed with Western blotting similarly to VP6-specific Western blotting. 1:2000 diluted monoclonal mouse anti-gp64 antibody IgG2b kappa (Santa Cruz Biotechnology, USA) and 1:10,000-diluted horse anti-mouse IgG-HRP (Vector Laboratories) were used in the analysis.

The production medium was purified similarly without the cell lysis step. Briefly, the medium was first ultracentrifuged at 100,000 g for 1.5 h at 4 °C and the resulting protein pellets were resuspended in 0.2 M Tris buffer pH 7.5 and incubated overnight at 4 °C. The resuspended pellets were then laid on top of “continuous” sucrose gradients and centrifugated at 100,000 g for three hours at 4 °C. After centrifugation, fractions were collected through bottom puncture and analysed by SDS-PAGE and VP6-specific Western blotting. Fractions containing the desired VP6 protein were combined and dialyzed against sterile PBS overnight.

### Production, purification and characterization of CVB3-VLPs

CVB3-VLPs were produced in High Five insect cells. First, our aim was to determine the optimal 3CD/P1 co-transfection ratio producing high level of high quality CVB3-VLPs. Therefore, we transfected the cells with 2%, 5% and 10% 3CD protease ratios in proportion to P1 polyprotein and analysed the effect of the production length on the VLP yield from 5- and 8-day productions. Extra- and intracellular VLP-protein fractions were collected from 40 ml productions by pelleting the cells and clarifying the production medium. The pellets were dissolved in 5 ml of 40 mM Tris pH 7.3, 10 mM MgCl_2_, 0.2 M NaCl, 0.1% Tween80 and the cells were lysed with sonicator (by repeating 2/5 s pulses (on/off) on ice, as with noro-VLPs). The VLP containing production medium was filtered through a 0.2 µm filter and the cleared supernatants and cell lysates were concentrated with 30% sucrose cushion ultracentrifugation as we have done previously^49^. For the 200 ml VLP production, VLP containing production medium was clarified by filtering the supernatant through a 0.2 µm filter. The VLP was purified with the same protocol we have used for the purification of BEVS produced CVB3-VLP previously^38^. Briefly, the VLP was concentrated by tangential flow filtration (TFF), utilizing 750,000 MWCO hollow fibre with an ÄKTA Flux system (Cytiva). The buffer was exchanged to 40 mM Tris pH 7.3, 10 mM MgCl_2_, 40 mM NaCl, and 0.1% Tween80 with the same system, and the impurities were removed with anion exchange chromatography and finally the VLPs were captured into SO_3_ cation exchange chromatography column and were eluted using NaCl gradient. VP1 and VP3 proteins were detected by Western blotting using in-house produced rabbit anti-CVB1-6 polyclonal antibody and IRDye-labelled secondary antibody (LI-COR Biosciences).

### Supplementary Information

Supplementary Information.

## Data Availability

The raw data used and/or analysed during this study are available from the corresponding authors upon reasonable request. The pOET5 plasmid sequences used in this study have been uploaded in Addgene (noro-VLP ID: 218092, rota-VLP ID: 218147, CVB3 ID: 218146) and the pOpie plasmid backbone sequence in GenBank (ID PP504784). For requests on plasmids to use in plasmid-based insect cell expression, please contact Maren Schubert (maren.schubert@tu-braunschweig.de).

## References

[1] Bachmann MF, Jennings GT (2010). Vaccine delivery: A matter of size, geometry, kinetics and molecular patterns. Nat. Rev. Immunol..

[2] Heinimaki S (2019). Combination of three virus-derived nanoparticles as a vaccine against enteric pathogens; enterovirus, norovirus and rotavirus. Vaccine.

[3] Hankaniemi MM (2019). Formalin treatment increases the stability and immunogenicity of coxsackievirus B1 VLP vaccine. Antiviral Res..

[4] Lampinen, V. *et al.* SpyTag/SpyCatcher display of influenza M2e peptide on norovirus-like particle provides stronger immunization than direct genetic fusion. *Front. Cell. Infect. Microbiol.* **13**, (2023). 10.3389/fcimb.2023.1216364 PMC10323135 37424789

[5] Haupt RM, Sings HL (2011). The efficacy and safety of the quadrivalent human papillomavirus 6/11/16/18 vaccine gardasil. J. Adolesc. Health Off. Publ. Soc. Adolesc. Med..

[6] Schiller JT, Castellsague X, Villa LL, Hildesheim A (2008). An update of prophylactic human papillomavirus L1 virus-like particle vaccine clinical trial results. Vaccine.

[7] Vicente T, Roldão A, Peixoto C, Carrondo MJT, Alves PM (2011). Large-scale production and purification of VLP-based vaccines. J. Invertebr. Pathol..

[8] Thompson CM, Aucoin MG, Kamen AA, Murhammer DW (2016). Production of Virus-Like Particles for Vaccination. Baculovirus and Insect Cell Expression Protocols.

[9] Correia R, Fernandes B, Alves PM, Carrondo MJT, Roldão A (2020). Improving influenza HA-Vlps production in insect high five cells via adaptive laboratory evolution. Vaccines.

[10] Yamaji H, Konishi E (2013). Production of Japanese encephalitis virus-like particles in insect cells. Bioengineered.

[11] Dai S, Zhang T, Zhang Y, Wang H, Deng F (2018). Zika virus baculovirus-expressed virus-like particles induce neutralizing antibodies in mice. Virol. Sin..

[12] Hong Q, Liu J, Wei Y, Wei X (2023). Application of baculovirus expression vector system (BEVS) in vaccine development. Vaccines.

[13] Carvalho SB (2016). Bioorthogonal strategy for bioprocessing of specific-site-functionalized enveloped influenza-virus-like particles. Bioconjug. Chem..

[14] Chaves LCS, Ribeiro BM, Blissard GW (2018). Production of GP64-free virus-like particles from baculovirus-infected insect cells. J. Gen. Virol..

[15] Klaus T (2015). Overcoming inefficient secretion of recombinant VEGF-C in baculovirus expression vector system by simple purification of the protein from cell lysate. Protein Expr. Purif..

[16] Yun E-Y (2005). Changes in cellular secretory processing during baculovirus infection. Biotechnol. Lett..

[17] Chen Y-R (2014). Transcriptome responses of the host trichoplusia ni to infection by the baculovirus Autographa californica multiple nucleopolyhedrovirus. J. Virol..

[18] Li T-C, Scotti PD, Miyamura T, Takeda N (2007). Latent infection of a new alphanodavirus in an insect cell line. J. Virol..

[19] Mena JA, Ramírez OT, Palomares LA (2007). Population kinetics during simultaneous infection of insect cells with two different recombinant baculoviruses for the production of rotavirus-like particles. BMC Biotechnol..

[20] Sokolenko S (2012). Co-expression vs. co-infection using baculovirus expression vectors in insect cell culture: Benefits and drawbacks. Biotechnol. Adv..

[21] Bieniossek C, Imasaki T, Takagi Y, Berger I (2012). MultiBac: Expanding the research toolbox for multiprotein complexes. Trends Biochem. Sci..

[22] Jaron M (2022). Baculovirus-free SARS-CoV-2 virus-like particle production in insect cells for rapid neutralization assessment. Viruses.

[23] Puente-Massaguer E (2020). PEI-mediated transient transfection of high five cells at bioreactor scale for HIV-1 VLP production. Nanomaterials.

[24] Korn J (2020). Baculovirus-free insect cell expression system for high yield antibody and antigen production. Sci. Rep..

[25] Rackoff LA, Bok K, Green KY, Kapikian AZ (2013). Epidemiology and evolution of rotaviruses and noroviruses from an archival WHO global study in children (1976–79) with implications for vaccine design. PLOS ONE.

[26] Atmar RL, Estes MK (2006). The Epidemiologic and clinical importance of norovirus infection. Gastroenterol. Clin. North Am..

[27] Koho T (2012). Purification of norovirus-like particles (VLPs) by ion exchange chromatography. J. Virol. Methods.

[28] Lampinen V (2021). Modular vaccine platform based on the norovirus-like particle. J. Nanobiotechnol..

[29] Blazevic V, Lappalainen S, Nurminen K, Huhti L, Vesikari T (2011). Norovirus VLPs and rotavirus VP6 protein as combined vaccine for childhood gastroenteritis. Vaccine.

[30] Tate JE (2012). 2008 estimate of worldwide rotavirus-associated mortality in children younger than 5 years before the introduction of universal rotavirus vaccination programmes: A systematic review and meta-analysis. Lancet Infect. Dis..

[31] Rotavirus vaccines: WHO position paper—July 2021. https://www.who.int/publications-detail-redirect/WHO-WER9628.

[32] Drory Y (1991). Sudden unexpected death in persons less than 40 years of age. Am. J. Cardiol..

[33] Alvarez JA, Wilkinson JD, Lipshultz SE (2007). Outcome predictors for pediatric dilated cardiomyopathy: A systematic review. Prog. Pediatr. Cardiol..

[34] Immunization Systems Management Group of the Global Polio Eradication Initiative. Introduction of inactivated poliovirus vaccine and switch from trivalent to bivalent oral poliovirus vaccine—worldwide, 2013–2016. *MMWR Morb. Mortal. Wkly. Rep.* **64**, 699–702 (2015). PMC4584682 26135591

[35] Hampton, L. M. Cessation of trivalent oral poliovirus vaccine and introduction of inactivated poliovirus vaccine—worldwide, 2016. *MMWR Morb. Mortal. Wkly. Rep.* **65**, (2016). 10.15585/mmwr.mm6535a3 27606675

[36] Enterovirus 71. https://www.who.int/teams/health-product-policy-and-standards/standards-and-specifications/vaccine-standardization/enterovirus-71.

[37] Koho T (2014). Coxsackievirus B3 VLPs purified by ion exchange chromatography elicit strong immune responses in mice. Antiviral Res..

[38] Hankaniemi MM (2020). Structural insight into CVB3-VLP non-adjuvanted vaccine. Microorganisms..

[39] Koho T (2015). His-tagged norovirus-like particles: A versatile platform for cellular delivery and surface display. Eur. J. Pharm. Biopharm..

[40] Heinimäki S (2022). Antigenicity and immunogenicity of HA2 and M2e influenza virus antigens conjugated to norovirus-like, VP1 capsid-based particles by the SpyTag/SpyCatcher technology. Virology.

[41] Lampinen V (2021). Modular vaccine platform based on the norovirus-like particle. J. Nanobiotechnology.

[42] Palomares LA, Ramirez OT (2009). Challenges for the production of virus-like particles in insect cells: The case of rotavirus-like particles. Biochem. Eng. J..

[43] Lappalainen S, Vesikari T, Blazevic V (2016). Simple and efficient ultrafiltration method for purification of rotavirus VP6 oligomeric proteins. Arch. Virol..

[44] Lepault J (2001). Structural polymorphism of the major capsid protein of rotavirus. Embo J..

[45] Tamminen K, Heinimaki S, Grohn S, Blazevic V (2020). Internalization and antigen presentation by mouse dendritic cells of rotavirus VP6 preparations differing in nanostructure. Mol. Immunol..

[46] Heinimäki S, Tamminen K, Hytönen VP, Malm M, Blazevic V (2020). Rotavirus inner capsid VP6 acts as an adjuvant in formulations with particulate antigens only. Vaccines.

[47] Hitchman RB (2010). Genetic modification of a baculovirus vector for increased expression in insect cells. Cell Biol. Toxicol..

[48] Krammer F (2010). Trichoplusia ni cells (High Five) are highly efficient for the production of influenza A virus-like particles: a comparison of two insect cell lines as production platforms for influenza vaccines. Mol. Biotechnol..

[49] Hankaniemi MM (2017). Optimized production and purification of Coxsackievirus B1 vaccine and its preclinical evaluation in a mouse model. Vaccine.

[50] Heinimäki S (2019). Combination of three virus-derived nanoparticles as a vaccine against enteric pathogens; enterovirus, norovirus and rotavirus. Vaccine.

[51] Bleckmann M (2019). Identifying parameters to improve the reproducibility of transient gene expression in High Five cells. PLoS ONE.

[52] Hausjell CS, Ernst W, Grünwald-Gruber C, Arcalis E, Grabherr R (2023). Quantitative proteomic analysis of extracellular vesicles in response to baculovirus infection of a Trichoplusia ni cell line. PloS One.

[53] Kato T, Yui M, Deo VK, Park EY (2015). Development of rous sarcoma virus-like particles displaying hCC49 scFv for specific targeted drug delivery to human colon carcinoma cells. Pharm. Res..

[54] Bleckmann M (2015). Genomic analysis and isolation of RNA polymerase II dependent promoters from Spodoptera frugiperda. PloS One.

[55] Rueden CT (2017). Image J2: ImageJ for the next generation of scientific image data. BMC Bioinformatics.

