# Supplementary material for: Production of norovirus-, rotavirus-, and enterovirus-like particles in insect cells is simplified by plasmid-based expression
Source: Sci Rep. 2024 Jun 27;14:14874. doi: 10.1038/s41598-024-65316-6 (PMC11211442; doi:10.1038/s41598-024-65316-6)
Supplement: Supplementary file 1 — Supplementary Information. [file 41598_2024_65316_MOESM1_ESM.docx]

***Supplementary information***

Plasmid-based production of noro, rota, and enterovirus-like particles in insect cells

Vili Lampinen^1,3^, Stina Gröhn^1^, Nina Lehmler^2^, Minne Jartti^1^, Vesa P. Hytönen^3,4^, *Maren Schubert^2†*^, *Minna M. Hankaniemi^1†*^

^1^Virology and Vaccine Immunology, Faculty of Medicine and Health Technology, Tampere University, Tampere, Finland

^2^Department of Biotechnology, Institute for Biochemistry, Biotechnology and Bioinformatics, TU Braunschweig, Braunschweig, Germany

^3^Protein Dynamics, Faculty of Medicine and Health Technology, Tampere University, Tampere, Finland

^4^Fimlab Laboratories, Tampere, Finland

† These authors contributed equally to this work and share last authorship

*** Correspondence:**Minna Hankaniemi
[minna.hankaniemi@tuni.fi](mailto:minna.hankaniemi@tuni.fi)

Maren Schubert
[maren.schubert@tu-braunschweig.de](mailto:maren.schubert@tu-braunschweig.de)

**List of supplementary information**

Supplementary figure 1

Supplementary figure 2

Supplementary figure 3

Supplementary figure 4

Supplementary figure 5

Supplementary figure 6


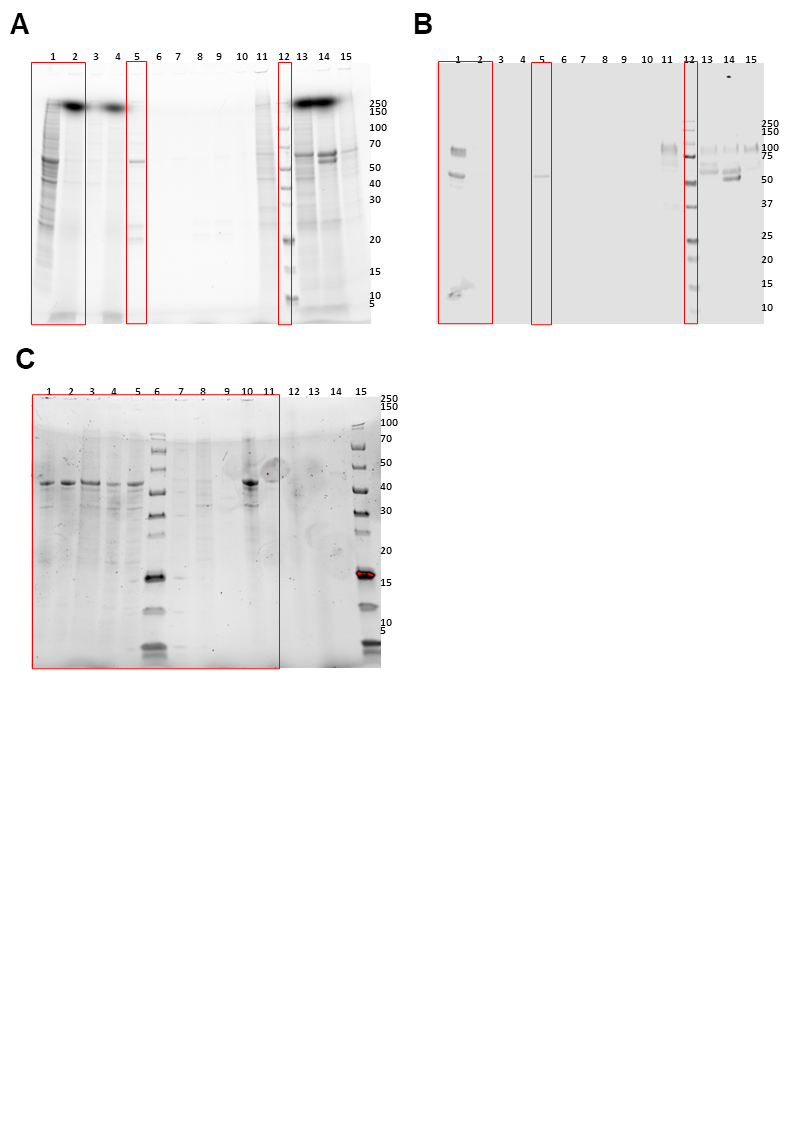


**Supplementary figure 1.** Optimization of plasmid-based noro-VLP expression A) Full electrophoresis gel cropped to figure 1A in three parts bordered with red. 1) Cells 2) Medium 3) Insoluble fraction from ultracentrifugation 4) Supernatant from ultracentrifugation 5) Ultracentrifuged 6–10) size exclusion chromatography fractions 11) Unrelated protein sample 12) Molecular weight marker 13–15) Unrelated protein samples B) Full Western blot membrane made from gel in panel A and cropped to figure 1B in three parts bordered with red. C) Full electrophoresis gel cropped to figure 1C. Lanes 1–5 are lysis samples from the soluble fractions and lanes 7–11 repeat the insoluble fractions in the same order. Lanes 13–15 contain unrelated protein samples. 1+7) Mild sonication 2+8) Strong sonication 3+9) French press 4+10) Detergent 5+11) Freeze/thaw 6+15) Molecular weight marker.

**
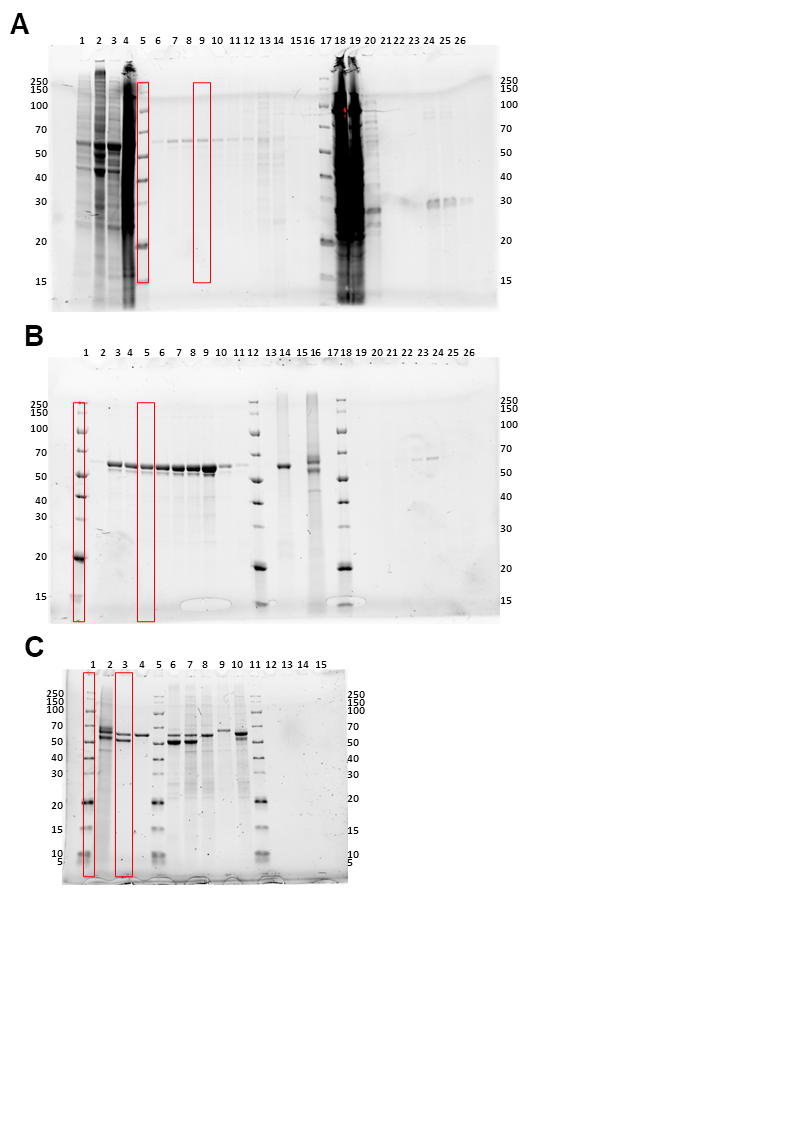
**

**Supplementary figure 2.** Characterisation of noro-VLP after purification. A) Full electrophoresis gel cropped to Figure 2A Transfection cells lane. 1) Cell fraction 2) Insoluble fraction after lysis 3) Soluble fraction after lysis 4) Expression medium 5+17) Molecular weight markers 6–11) Pooled noro-VLP fractions from size exclusion chromatography 12–16) Discarded fractions 18–26) Unrelated protein samples B) Full electrophoresis gel cropped to Figure 2A Transfection medium lane. 1+12+18) Molecular weight markers 2–4) Discarded size exclusion chromatography fractions 5–10) Pooled noro-VLP fractions from size exlusion chromatography 11) Discarded fraction 13–17) Unrelated protein samples 19–26) Unrelated protein samples C) Full electrophoresis gel cropped to Figure 2A BEVS medium lane. 1+5+11) Molecular weight markers 2) Unrelated protein sample 3) Purified noro-VLP from baculovirus expression system 4) Unrelated protein sample 6–10) Unrelated protein samples 12–15) Empty wells

**
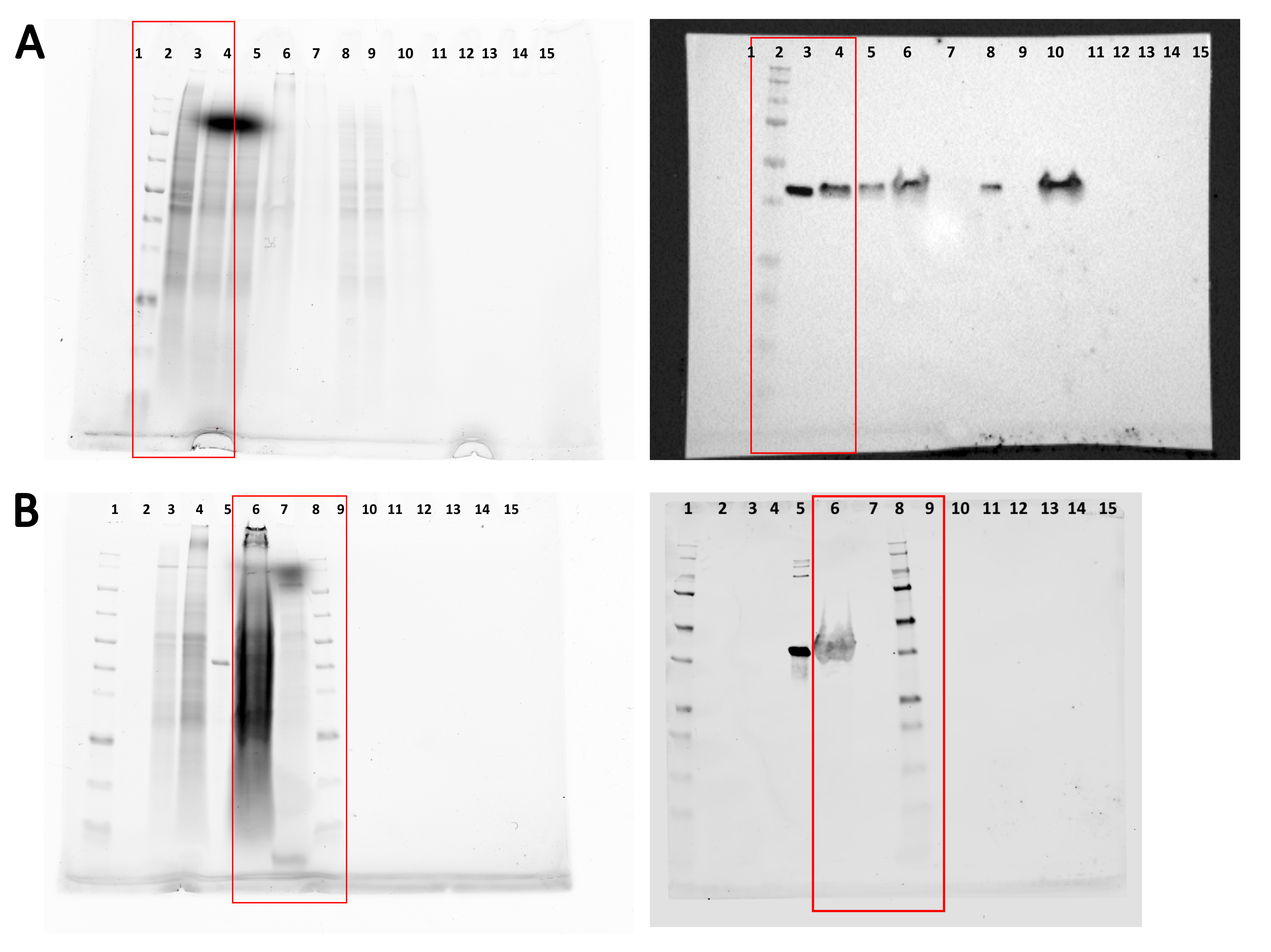
**

**Supplementary figure 3.** Accumulation compartments of VP6 produced in insect cells with BEVS or plasmid-base system. A) Full SDS-PAGE gel (left panel) and Western blot images (right panel) cropped to Figure 3A SDS-PAGE and Western blot analyses of VP6 produced with BEVS. 15 µl of each sample was loaded per well as follows 1) Empty well 2) Molecular weight marker 3) intracellular BEVS-based sample 4) extracellular BEVS-based sample 5–10) Unrelated protein samples and 11-15) empty wells B) Full SDS-PAGE gel (left panel) and Western blot images (right panel) cropped to Figure 3B SDS-PAGE and Western blot analyses of VP6 produced with plasmid-based system. 15 µl of each sample was loaded per well as follows 1) Molecular weight marker 2-5) Unrelated protein samples 6) intracellular plasmid-based sample 7) extracellular plasmid-based sample 8) Molecular weight marker and 9-15) Empty wells. Red boxes indicate the areas cropped for Figure 3A and B.


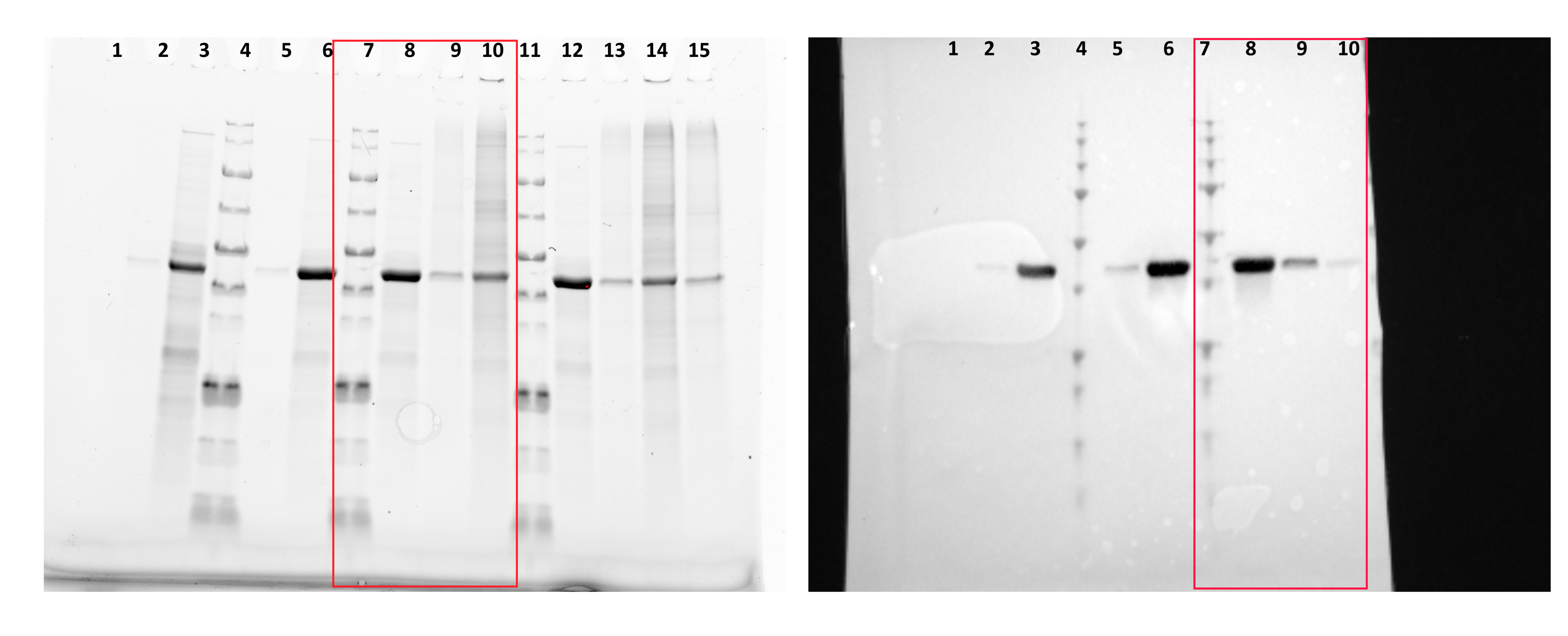


**Supplementary figure 4.** Characterization of VP6 nanostructures after purification. The left panel shows the full SDS-PAGE gel image cropped to Figure 4A left panel and the right panel the Western blot image cropped to Figure 4A right panel. 15 µl of each purified VP6 was loaded per well as follows 1) Empty well 2-3) Unrelated protein samples 4) Molecular weight marker 5-6) Unrelated protein samples 7) Molecular weight marker 8) Intracellular plasmid-based VP6 (c = 0.262mg/ml, loaded 3.93µg) 9) BEVS-based intracellular VP6 (c = 0.094mg/ml, loaded 1.41µg) 10) BEVS-based extracellular VP6 (c = 0.277mg/ml, loaded 4.16µg) 11) Molecular weight marker and 12-15) Unrelated protein samples. Red boxes indicate the areas cropped for Figure 4A.

**
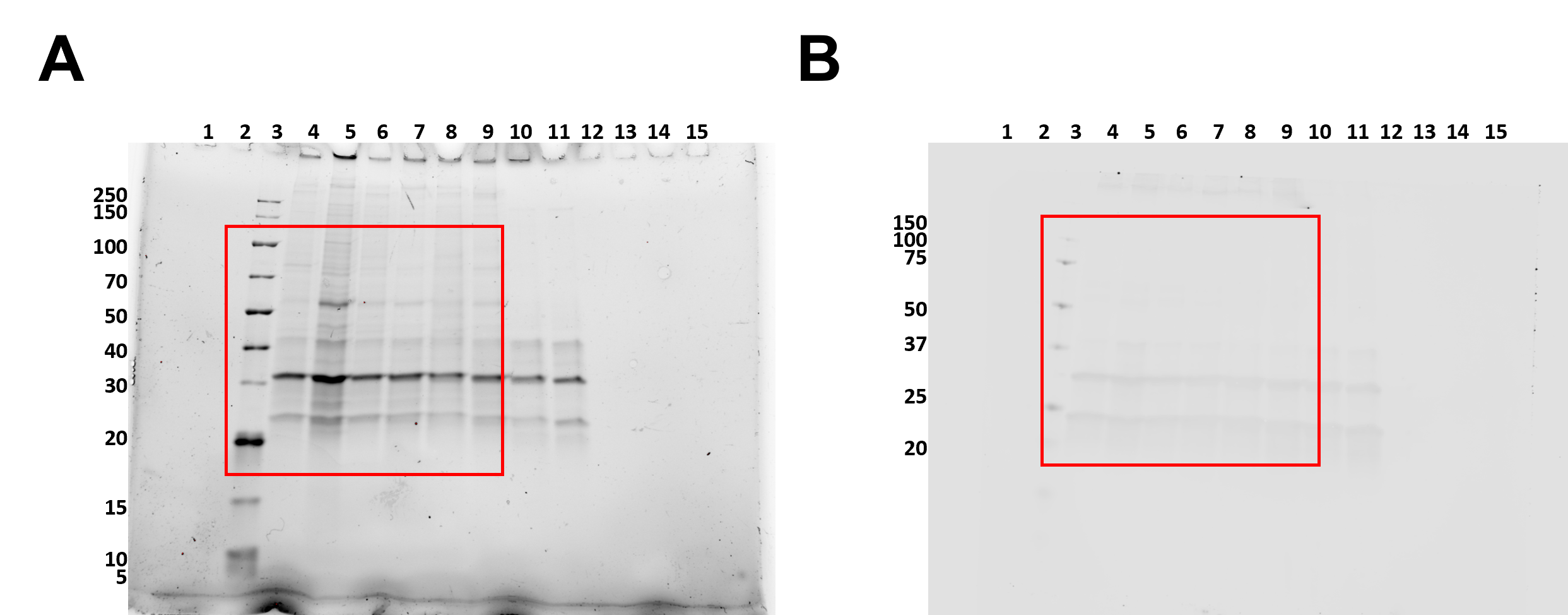
Supplementary figure 5.** Characterisation of CVB3-VLPs produced by co-transfection of 3CD and P1. A) SDS-PAGE and B) Western blot analyses of purified VLPs. Full electrophoresis gel (A) and Western Blot (B) made from the gel were cropped in figure 5. 1–2) Empty wells 3) Molecular weight marker 4) 2 % 3CD/98 % P1, 5 days production 5) 2 % 3CD/98 % P1, 8 days production 6) 5 % 3CD/95 % P1, 5 days production 7) 5 % 3CD/95 % P1, 8 days production 8) 10 % 3CD/90 % P1, 5 days production 9) 10 % 3CD/90 % P1, 8 days production 10-11) see supplementary figure 6 12–15) Empty wells.


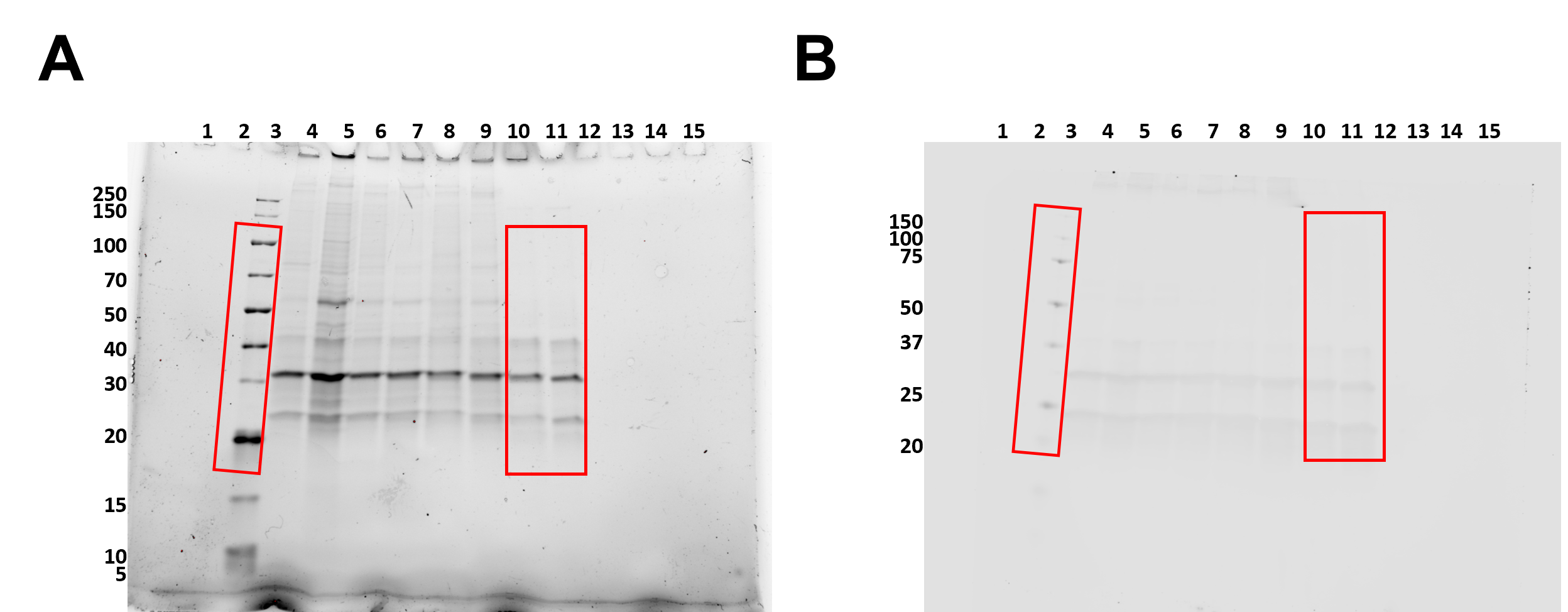


**Supplementary figure 6.** Characterisation of CVB3-VLP after large-scale production and ion-exchange purification. Full electrophoresis gel (A) and Western Blot (B) made from the gel were cropped in figure 6. 1–2) Empty wells 3) Molecular weight marker 4-9) see supplementary figure 5 10) plasmid-based system 10 % 3CD/90 % P1, 8 days production and 11) baculovirus expression system, 5 days production.
